# Royal jelly accelerates healing of acetate induced gastric ulcers in male rats 

**Published:** 2020

**Authors:** Mohammad Sofiabadi, Fatemeh Samiee-Rad

**Affiliations:** 1 *Cellular and Molecular Research Center, Qazvin University of Medical Sciences, Qazvin, Iran*; 2 *Metabolic Diseases Research Center, Qazvin University of Medical Sciences, Qazvin, Iran*

**Keywords:** Royal jelly, Gastric ulcer, Rat

## Abstract

**Aim:**

This study examined the healing potential of royal jelly on the acetic acid induced wounds healing in male rat’s gastric mucosa.

**Background:**

Scientific reports suggest that, bee products can help in the wounds healing.

**Methods:**

96 adult male Wistar rats were divided into in 4 groups as follows: control, omeprazole 20 mg/kg, and royal jelly 50 and 200 mg/kg). Wound was induced in stomach mucosa of each rat with 100% acetic acid. Samples groups received omeprazole or royal jelly from 1st to 14th day after acetic ulcer induction. Gastric ulcer healing and histopathological parameters were evaluated on 4, 7, 10, 15th days after ulceration. Both descriptive and statistical analyses were used. P <0.05 was considered as significant.

**Results:**

The royal jelly administration significantly reduced the depth of lesion in comparison with the control group (p<0.05) and attuned histopathological changes in the treatment groups. The largest healing effect was demonstrated with royal jelly on 10th treatment day, at a higher concentration (200 mg/kg).

**Conclusion:**

These findings supported that royal jelly had effectively contributed to the wound healing, valid gastroprotective activity, and can be used for peptic ulcer therapy.

## Introduction

 Beehive products such as honey, propolis, and royal jelly (RJ) are attractive ingredients of healthy foods. These crops have been used since ancient times as a part of traditional medicine. Royal jelly has a complex composition of proteins, fats, fatty acids, free amino acids, organic acids, sterols, phenols, sugars, mineral salts, vitamins, and other unknown substances, which was reported that some of these compounds can reduce the inflammatory response and infectious process and improve the tissues blood circulation (1). Previous research demonstrated that the royal jelly antioxidant property can be associated with the biological activity of free amino acids (2).

The more beneficial effects of royal jelly are mainly attributed to the phenolic compounds such as flavonoids. Flavonoids play role on the activity of some enzymes including cyclo-oxygenase and lipoxygenase (1). Royal jelly also regulated the balance between the pro-oxidative and antioxidative effectors (3). 

Royal Jelly has been considered and used since old times for health, and it is still very important in Asian traditional and folkloristic medicine. Royal Jelly is honeybee hypopharyngeal gland secretion of young worker bee, and is an exclusive nourishment for bee queen. Recently, RJ and its components have been re-subjected to extensive usage for several investigations. Also, it was reported that the crude royal jelly or its major proteins such as royalisin, 10-hydroxy-2-decenoic acid, and jelleines show a highest effect on different types of bacteria, especially on Gram positive bacteria (4).

Gastric and duodenal ulcers can be both defined as peptic ulcer disease, and globally is a prevalent problem and responsible for substantial premature mortality (5). Peptic ulcer disease is a penetrate defect beyond the muscularis mucosae of the gastric or duodenal wall, and its complications can include upper gastrointestinal bleeding, perforation, and rarely gastric outlet obstruction (6). Also, the main peptic ulcer symptoms are epigastric pain and heartburn (5). 

Many pharmacological and non-pharmacological treatments used for the prevention of peptic ulcer disease and healing of the mucosal damage. Drugs such as antacids, proton-pump inhibitors (PPIs), prostaglandin analogues, histamine-2 receptor antagonists (H2RAs), and anticholinergics have some complications. Drugs used to prevent and treat gastric ulcers should have potent therapeutic effect as well as rare complications (7).

Also, many plants and herbal derived products had antiulcerogenic potential activity via ulcer healing, antioxidant, anti-inflammatory, cytoprotective, gastric secretion inhibition, mucus production improvement, anti-secretory, and anti-H. Pylori activities (8-10). 

The previously described beneficial effects of Royal Jelly could be effective on the treatment of peptic ulcer, Accordingly, Belostotskiĭ et al. (2009) revealed that, the administration of bee-keeping products can decrease the stomach acid secretion and accelerate gastric ulcers healing in the rats (11). 

In this relationship, de Barros et al. (2006) showed that, treatment with propolis (a resinous substance of honey bees) can significantly reduce the gastric volume, total acidity, pH, and gastric lesion induced by ethanol or indomethacin (12). 

The gastric mucosa is under the continuous detrimental physical and chemical states such as daily exposure to acid and pepsin coupled with a wide variety of potentially damaging agents such as certain foods, a range of temperatures, hyperosmolar and abrasive substances, refluxed bile and pancreatic juice, bacterial toxins, and damaging drugs. However, the gastric mucosa integrity is retaining via gastric mucus barrier activity (13). 

Royal Jelly usage recommended by modern herbalists due to having antibacterial, protective, anti-inflammatory, antioxidative, immunomodulatory, and anti-ulcer properties (13, 14).

According to several therapeutic effects of RJ such as antimicrobial, vascular relaxant, growth stimulant, and anti-inflammatory activity, it is suggested for treating the wounds. Also, royal Jelly has low costs and lesser adverse reactions compared to traditional therapeutic methods (1).

 So, the present study aimed to evaluate the healing potential of royal jelly on the acetic acid induced wounds healing in the male rat’s gastric mucosa. 

## Methods

**Animals**

96 albino Wistar rats (200.00±2 g) were obtained from razi institute (Karaj- Iran) and reared as described by Sabiu et al. (15). They were kept at room temperature under the natural day/night cycles. They were fed with a standard laboratory diet (supplied by pars Ltd, Karaj, Iran) and were given tap water ad libitum. Then, the approval was obtained from the Ethical Committee on the Use and Care of Laboratory Animals of the Qazvin University of Medical science Qazvin, Iran, with a certified number RES.IR. QUMS.REC.1394.121. All the protocols used in this study conformed to the ethical guidelines of 1975 Helsinki Declaration.

**Induction of gastric ulcers**

We applied the method defined by Pillai (16) and Wang et al. (17) with small modifications. 

Induction of gastric ulcers via this model was simple, popular, and exactly produced deep sited gastric ulcer in animal models. 

In that way, after 24-hour fasting, upper abdomen of rats was opened under the ether anesthesia. Then, 50 µl of 30% glacial acetic acid was injected into the wall of the stomach corpus at the region of the lesser curvature, and the stomach wall was washed using cotton soaked in a 0.9% NaCl solution. Then, the fascia and skin of the cutting region were sutured. Daily, the skin was disinfected by iodine. Also, the routine feeding was continued after surgery.

**Main procedure**

By passing one day from the acetic ulcer induction; animals were randomly divided into 4 groups (n=96) as follows: control (normal saline), omeprazole (as reference drug, 20 mg/kg), and two royal jelly (50 and 200 mg/kg) treatment groups. All administrations were performed orally by the use of orogastric tube with 0.2 ml volume from the 1st to 14th day. Rats had free access to food and water during the trial period. Royal Jelly was collected from the colonies of South Qazvin bee colonies. Normal saline was used as a royal jelly and omeprazole vehicle.

**Preparation of histological section and examination**

At intervals in 4, 7, 10, and 15th days after induction of ulcers; the animals were anesthetized by inhalation of the ether, and the stomach was carefully removed. The stomach was incised along with the greater curvature and washed with normal saline, and was then transported into 10% formalin solution for performing histological study. The sections of gastric wall were perpendicular to the surface of each wound. Slides were stained by H & E method, and were microscopically detected at the high magnification (×400) for blood vessels (Three fields were selected. After vascular count, the mean was determined) and×1,000, oil emersion, for PMN leukocytes and fibroblasts (Four fields were selected. After cells count, the mean was determined) (18).

All slides were observed by Cellsens Entry software. The associated images were taken by the Olympus DP25 camera. Then, the numbers of PMN cells, fibroblasts, and new blood vessels were counted. To assess the epithelization, we used modified scoring system of the wound tissue histological features, which was previously described by Gal et al. (19)

As following form: 0: No epithelial organization, 1: mimimal epithelial organization, 2: mild epithelial organization, 3: moderate epithelial organization, and 4: complete epithelial organization. All histopathologic examinations were performed by a pathologist who was blinded to the treatment groups.

**Statistical analysis**

Values in tables are given as arithmetic means ± standard error of the mean (SEM). The collected data were analyzed using the Statistical Package for the Social Sciences (SPSS) software (version 21.0, SPSS Inc, Chicago, IL USA). Data were described as mean ± standard error of the mean. Multiple comparisons were made among and within each group of rats at intervals. The significance degree of differences among these groups was analyzed by the means of Analysis of Variance (ANOVA) followed by Tukey post hoc test. A level of P<0.05 was accepted as statistically significant. 

## Results

The healing of acetic ulcers was accelerated with the administration of royal jelly or omeprazole during 14 days. The largest healing effect was demonstrated with royal jelly at the dosage of 200 mg/kg. A decrease of stomach wound depth was revealed in the rats, which have received royal jelly versus the rats of control group.

**Histological parameters**

The histological demonstration of the stomachs subjected to “gastric ulcers” is shown in Table 1.2. The administration of royal jelly in both doses (50,200 mg/kg) or omeprazole (20 mg/kg) was shown a reduction in the number of PMN leukocytes in comparison with the control group, which was significant at days 4, 7, and 10th-day (p <0.01), which on 7 and 10th-day, the effect of royal jelly on 200 mg/kg dosage was greater than the omeprazole (p<0.05)

Also, the daily administration of omeprazole or royal jelly (dose- dependent) significantly increased the stomach wounds fibroblasts, especially on the 10th day of the test, as compared to the control (p <0.01). However, at the 15th test day, the difference between the all groups was not statistically significant.

The number of new vessels significantly increased in the omeprazole or royal jelly groups compared to the control (p<0.01). In this parameter, at the 7th day, the royal jelly in 200 mg/kg dosage was more effective than omeprazole (p<0.05). (Table 1). 

**Ulcer healing**

The Microscopic aspect of gastric ulcers is shown in Tables 1 and 2. Prescription of Royal Jelly or omeprazole accelerated the wound healing process; and consequently, the depth of wound in the treated groups, especially on the 4th and 15th days, was lower than the control group (p<0.001). (Table 2).The healing parameters including depth and duration of complete healing showed significant difference among the treatment groups and control group (p <0.001).

**Histological presentation**

**Table 1:** Effects of royal jelly and omepersole on gastric histopathologic and histomorphometric of acetic ulcerated rats (Mean±SD)

Histopathological analysis	Control (n=6)	Omeprazole (n=6)	Jel50 (n=6)	Jel200 (n=6)
Day 4				
PMNs	78±9.72	67.5±3.22	61.5±1.32	47.75±1.02 b
Fibroblast	20.75±2.13	23.75±1.98	25±1.22	29.25±1.49 a
Vascular no.	9.75±1.49	12.75±1.18	12.75±0.96	18±0.82 b
Epithelization	0.00	0.00	0.00	1
Day 7				
PMNs	43.25±2.65	34.75±1.88	32.75±2.28	20.5±1.65 b c
Fibroblast	29.00±3.02	41.25±2.89	37.75±1.95	52.25±1.43b
Vascular no.	16.5±1.70	26±1.52a	25.5±2.08a	26.25±.91b
Epithelization	1	1	1	2
Day 10				
PMNs	24.75±2.05	18.5±1.93a	12.5±1.44a	8.75±3.02 b
Fibroblast	31.25±1.68	44.25±2.39	44.5±2.29a	53.75±4.87b
Vascular no.	22.75±1.75	33. 25±2.32a	33.75±2.73a	36.75±3.02a
Epithelization	2	3	3	3
Day 15				
PMNs	7.60±0.8	4.5±0.9 b	3.75±0.8	4.25±0. 9 a
Fibroblast	37.75±4.16	49.25±1.88	46.5±3.15	48.75±1.75
Vascular no.	14.75±1.75	21.25±3.22	22.75±1.67	28.00±1.29a
Epithelization	3	4	4	4

**a **Significantly different from the control group (p<0.05).

**b **Significantly different from the control group (p<0.01).

**C **Significantly different from the omeprazole group (p<0.05).

Omeprazole (20 mg/kg); RJ, Royal Jelly (50, 200 mg/kg); Depth of ulcer: Micrometer

**Table 2:** Effects of royal jelly and omepersole on the gastric ulcer healing of acetic ulcerated rats (Mean±SD)

Ulcer healing	Control	Omeprazole	Jel50	Jel200
Day 4				
Depth of ulcer (µm)	812.5±23.93	712.5±23.56a	700±20.41a	625±14.43b
Day 7				
Depth of ulcer	587.5±23.43	412.5±31.45a	425±20.41a	405±14.43b
Day 10				
Depth of ulcer	425±14.43	325±14.62a	317.5±11.93	240±13.54b
Day 15				
Depth of ulcer	150±12.43	63.75±13.67b	64.5±12.81b	37.5±9.68b

**a **Significantly different from the control group (p<0.05).

**b **Significantly different from the control group (p<0.01).

Omeprazole (20 mg/kg); RJ, Royal Jelly (50, 200 mg/kg); Depth of ulcer: Micrometer

**Figure 1:**
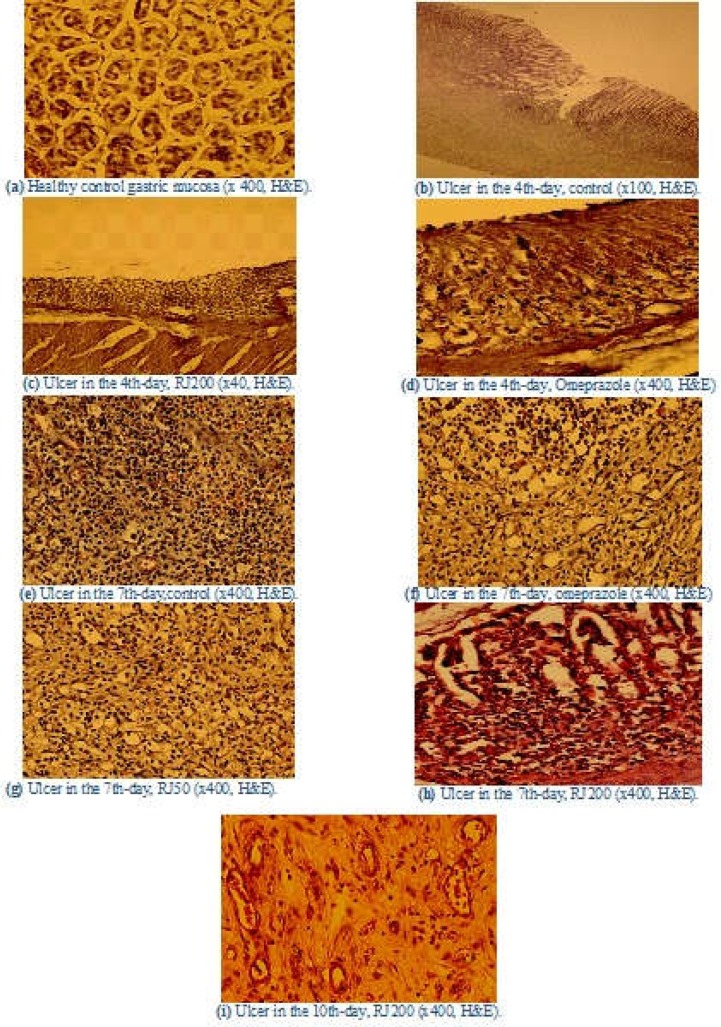
Histopathologic findings of rat’s gastric ulcer healing in different groups. (a) Normal stomach mucosa of control non -ulcerated rats (x 400). (b) Deep sited gastric ulcer in the 4th-day of control's rats (x100). (c) Gastric mucosa in RJ 200 treated rats showed the loss of superficial epithelium and sloughed glandular epithelial cells (x40). (d) Stomach mucosa in Omeprazole treated group showed mucosal necrosis(x400). (e,f,g,h) Ulcer in the 7th-day gastric ulcer healing in control, omeprazole, RJ50, and RJ200 groups rats, respectively(x400). (i) Granulation tissue organization, fibroblastic proliferation, and collagen deposition in the 10th-day gastric ulcer healing in RJ200 group rats (x400)

The histopathologic findings of gastric ulcer repair are exhibited in figure 1. In the 4th-day control, compared with the healthy gastric mucosa; histological observation showed deep sited mucosal ulcer (a,b). RJ 200 treated group showed the loss of superficial epithelium, and many glands had sloughed glandular epithelial cells lying loosely in the gland lumen, and many inflammatory cells could be observed in the interstitial tissue (c). Also, omeprazole treated group showed mucosal necrosis (d).

Inflammation, granulation tissue formation, and organization with collagen deposition were also observed, but only in the groups that were treated with RJ or omeprazole, and these changes were significantly lower than the control group (p <0.01)(e, f, g, h, i in figure 1). Also, in the 7, 10, and 15th days, the organization of granulation tissue, density of fibroblasts, and collagen fiber deposition in the treated groups were greater than the control (p<0.01). The epithelization of the central wound area was nearly completed at the 14th day in the royal jelly group.

## Discussion

In the present study, the healing effects of RJ were evaluated on acetic acid induced gastric ulcers. Also, the histological data revealed that, the oral administration of RJ significantly accelerated the healing of acetic-acid-induced gastric ulcers compared to the control. In agreement with our result, the previous studies have also shown the healing activities of the RJ on different ulcers (20). 

The experimental model of acetic acid easily induced ulcers and reliably produced highly look like human stomach ulcers in point of pathophysiology and the associated underlying pathways involved in wound healing. These ulcers are mainly due to the corrosive action of acid and part of it, is due to increased gastric secretion in response to stress generated by pain resulted from abdominal surgery (21). 

 This increase contains back diffusion of the H+ ions through the surface mucus layer, toward the internal layers of the gastric wall. These consequences can result in tissue failure, which triggers the release of arachidonic acid metabolites and absorbs polymorphonuclear (PMN) leukocytes that can stimulate inflammation and superficial injury, and extended into deeper mucosal to cause mucus lesions and inactivate growth factors, which are necessary for mucosal integrity and repairing (22, 23). 

The phenolic compounds of RJ induce synthesis of prostaglandin E2 (PGE2), which is in contrary with the declined mucin liked glycoproteins, via mucus secretion activities (24). The wound repair is well organized cascade, which is separated into four overlapping stages including hemostasis, inflammation, proliferation, and remodeling (25).

 In this study, a high degree of ulceration (812.5 ± 23.93 μm) was observed in the control group by passing 4 days from the acetic acid ulcer induction. Previous studies showed that, in control animals, ulcer size were reduced by auto recovery during the couple of weeks (26). This auto treatment is due to the mucosal damage, which constructively and effectively stimulates the adjacent mucosa and ulcer bed, to secret growth factors (27).

 Treatment with Royal jelly greatly accelerated wound healing, which was stronger than omeprazole effect at some intervals. Previous studies showed that the suppression of endogenous prostaglandins reasons of healing deletion through excessive cytokine expression and release. This resulting basal gastric acid secretions increased and the mucosal defensive mechanism weakened. So, the weakened ulcerated area resulting in delayed ulcer healing (27, 28). 

The stomach uses two mechanisms to protect itself against acid and pepsin. Accordingly, one of them is controlling the amounts of acid and pepsin productions through hormonal and neurological feedback, and the proportional productions of mucus and bicarbonate. If the balance between the two is eliminated; for example, by inhibiting the production of prostaglandins, it will weaken the gastric defensive barrier. Furthermore, the gastric mucosal protection can be strengthened by reducing direct physical contact between the stomach juice and gastric epithelium by the isolation of bicarbonate and pH slope between the gastric epithelium and its lumen. These protective phonemes will also facilitate the recovery process of epithelial damages (29).

 The creation of induced gastric ulcers is a Multifactorial event, in which the mucus content depletion is the first step (30). Therefore, epithelial cells are susceptive to cell injury, and excessive production of free radicals is initiating (31).

 Free radicals induce reversible and irreversible cell injury and cell death (32). The RJ regulated the balance between the pro-oxidative and anti-oxidative effectors and has antioxidant property; therefore, the administration of RJ created interference with excessive oxidative cascade and production of free radicals (2, 3). 

At the other hand, reactive oxygen species (ROS) play role on the pathogenesis and generation of gastric ulcers, which occur as a result of combined actions of abdominal surgery (33) and acetic acid injection (34). It is now known that, ROS hyper production involve gastrointestinal inflammation and gastric ulcer (35). It is known that, oxidative stress is one of the major pathogens that can directly affect the oxidative damages including lipid peroxidation, protein oxidation, and DNA damage, which can lead to cell death (36). 

So, RJ due to its anti-inflammatory and anti-oxidant effects, can reverse this condition and facilitate ulcer healing. In this regard, according to available research, royal jelly has an effective antioxidant property and an inhibition capacity for free radicals (36). For example, it has been shown that, royal jelly can inhibit in vivo and in vitro peroxidase enzyme and protect DNA from oxidative damage (37).

Histological sections of rat stomachs illustrate the damage caused by acetic acid, as well as the healing process induced by the RJ or omeprazole. Merely like that, the administration of omeprazole can significantly inhibit both basal and histamine-stimulated gastric secretions in the rats with ulcers, which lead to declined gastric acidity, declined acid output, and declined stomach volume; therefore, RJ may have the same mechanism or perform in a similar way (38). 

It has been reported that, the Royal Jelly appears to have a stimulant effect on nerve growth, and facilitates the differentiation of neurons from stem / progenitor cells. One of the unique compounds of royal jelly is a hydroxydicanoic unsaturated Trans fatty acid, which may act as a neurotrophic factor. This compound may possibly have this proliferative effect on gastric mucosa (39). 

During healing, the granulation tissue undergoes continuous remodeling, whereby the inflammatory cells that was appeared in the early phase of healing, are replaced by fibroblasts and micro vessels in the late healing phase (38). 

Our results showed that, RJ could effectively decrease PMN leukocyte resulting in attenuate inflammations, and at this time increase the fibroblast and new microvascular, which all of these conditions cam promote ulcer healing and ulcer re-epithelialization. These processes are mediated by growth factors, so it may be RJ that can stimulate productions by platelets, injured tissue, and macrophages to restore the continuity of the epithelial lining, which is essential for ulcer healing since it generates a barrier protecting the granulation tissue from any mechanical and chemical damages (40).

 Other hypothetical mechanisms, in addition to the increases of mucus production and antioxidant levels, on the one hand may include the decrease of gastric acid secretion, all these factors taken together can develop angiogenesis, cellular proliferation, cellular migration, and granulation tissue maturation (37). 

In agreement with our clarifications, Belostotskiy et al. reported that, administration of several honey bee products including royal jelly can decrease acid secretion and pepsin activity and accelerate the recovery of gastric ulcers (11). 

Also, the results of de Barros et al. and El-Hady studies showed that the administration propolis significantly reduced the number and depth gastric ulcer via cytoprotective activity; raised the mucus and/or prostaglandins generation; and diminished the acid secretion volum, acidity, and anti-histaminic effect (12, 41). 

The findings of Czarnecki et al. revealed that, the treatment with propolis accelerated gastric ulcer repair via considerable decrease on the ulcer number and surface area. These findings supported our results (42). 

Based the antibacterial activity of RJ , another mechanism of antiulcer effects was suggested, but further evaluation of this antibacterial effect in relation to inhibiting the growth of Helicobacter pylori (a major cause of ulcer) are required (43). 

In addition to antibacterial effect of RJ, it also contains enzymatic and vasodilate properties, and it can potentially help the healing of ulcers (44). 

The results of the present study suggest that, RJ can be a useful resource for the production of a standardized antiulcer product, especially given that toxicological studies have demonstrated the innocuousness of this natural substance. Thus, the use of this natural product could be recommended for the management of chronic gastric ulcers for the patients who have hyper acid secretion gastric ulcer such as gastrinoma.

## References

[1] Viuda-Martos M, Ruiz-Navajas Y, Fernández-López J, Pérez-Alvarez JA (2008). Functional properties of honey, propolis, and royal jelly. J Food Sci.

[2] Silici S, Ekmekcioglu O, Eraslan G, Demirtas A (2009). Antioxidative effect of royal jelly in cisplatin-induced testes damage. Urology.

[3] Kocot J, Kiełczykowska M, Luchowska-Kocot D, Kurzepa J, Musik I (2018). Antioxidant Potential of Propolis, Bee Pollen, and Royal Jelly: Possible Medical Application. Oxid Med Cell Longev.

[4] Fratini F, Cilia G, Mancini S, Felicioli A (2016). Royal Jelly: An ancient remedy with remarkable antibacterial properties. Microbiol Res.

[5] Sayehmiri K, Abangah G, Kalvandi G, Tavan H, Aazami S (2018). Prevalence of peptic ulcer in Iran: systematic review and meta-analysis methods. J Res Med Sci.

[6] Tonolini M, Ierardi AM, Bracchi E, Magistrelli P, Vella A, Carrafiello G (2017). Non-perforated peptic ulcer disease: multidetector CT findings, complications, and differential diagnosis. Insights Imaging..

[7] Kuipers EJ (2018). PPIs for prevention and treatment of peptic ulcer. Lancet Gastroenterol Hepatol.

[8] Toma W, Hiruma-Lima CA, Guerrero RO, Brito A (2005). Preliminary studies of Mammea americana L (Guttiferae) bark/latex extract point to an effective antiulcer effect on gastric ulcer models in mice. Phytomedicine.

[9] Sidahmed HM, Hashim NM, Mohan S, Abdelwahab SI, Taha MM, Dehghan F (2016). Evidence of the gastroprotective and anti-Helicobacter pylori activities of β-mangostin isolated from Cratoxylum arborescens (vahl) blume. Drug Des Dev Ther.

[10] Sidahmed H, Abdelwahab SI, Mohan S, Abdulla MA, Mohamed Elhassan Taha M, Hashim NM (2013). α-Mangostin from Cratoxylum arborescens (Vahl) Blume demonstrates anti-ulcerogenic property: a mechanistic study. Evid Based Complement Alternat Med.

[11] Belostotskiĭ NI, Kas'ianenko VI, Dubtsova EA, Lazebnik LB (2009). Influence of honey, royal jelly and propolis on accelerating acetate healing of experimental gastric ulcers in rats. Eksp Klin Gastroenterol.

[12] de Barros MP, Sousa JP, Bastos JK, de Andrade SF (2007). Effect of Brazilian green propolis on experimental gastric ulcers in rats. J Ethnopharmacol.

[13] Haruna L, Aber A, Rashid F, Barreca M (2012). Acute mesenteric ischemia and duodenal ulcer perforation: a unique double pathology. BMC Surg.

[14] Kanbur M, Eraslan G, Silici S, Karabacak M (2009). Effects of sodium fluoride exposure on some biochemical parameters in mice: Evaluation of the ameliorative effect of royal jelly applications on these parameters. Food Chem Toxicol.

[15] Sabiu S, Wudil AM, Sunmonu TO (2014). Compined administration of Telfaira occidentalis and Vernonia amygdalina leaf powder ameliorates garlic –induced hepatotoxicity in Wistar rats. Pharmacologia.

[16] Pillai NR, Santhakumari G (1984). Effects of nimbidin on acute and chronic gastro-duodenal ulcer models in experimental animals. Planta Medica.

[17] Wang JY, Hitoshi N, Susumu O (1990). Effect of omeprazole on delayed healing of acetic acid-induced gastric ulcers in rats. Jpn J Pharmacol.

[18] Talas D, Nayci A, Atis S, Polat A, Comelekoglu U, Bagdatoglu C (2002). The effects of corticosteroids on the healing of tracheal anastomoses in a rat model. Pharmacol Res.

[19] Gál P, Toporcer T, Vidinský B, Mokrý M, Grendel T, Novotný M, Novotný J (2008). et al. Postsurgical administration of estradiol benzoate decreases tensile strength of healing skin wounds in ovariectomized rats. J Surg Res.

[20] Belostotskiĭ NI, Kas'ianenko VI, Dubtsova EA, Lazebnik LB (2009). Influence of honey, royal jelly and propolis on accelerating acetate healing of experimental gastric ulcers in rats. Eksp Klin Gastroenterol.

[21] Amagase K, Yokota M, Tsukimi Y, Okabe S (2003). Characterization of "unhealed gastric ulcers" produced with chronic exposure of acetic acid ulcers to indomethacin in rats. J Physiol Pharmacol.

[22] Johansson M, Synnerstad I, Holm L (2000). Acid transport through channels in the mucous layer of rat stomach. Gastroenterology.

[23] Schubert ML, Peura DA (2008). Control of gastric acid secretion in health and disease. Gastroenterology.

[24] Alanko J, Riutta A, Holm P, Mucha I, Vapaatalo H, Metsä-Ketelä T (1999). Modulation of arachidonic acid metabolism by phenols: relation to their structure and antioxidant/prooxidant properties. Free Radic Biol Med.

[25] Abreu AM, Oliveira DW, Marinho SA, Lima NL, de Miranda JL, Verli FD (2012). Effect of topical application of different substances on fibroplasia in cutaneous surgical wounds. ISRN Dermatol.

[26] Schmassmann A (1998). Mechanisms of ulcer healing and effects of nonsteroidal anti-inflammatory drugs. Am J Med.

[27] Brzozowski T, Konturek PC, Konturek SJ, Schuppan D, Drozdowicz D, Kwiecień S (2001). Effect of local application of growth factors on gastric ulcer healing and mucosal expression of cyclooxygenase-1 and -2. Digestion.

[28] Wallace JL (2008). Prostaglandins, NSAIDs, and gastric mucosal protection: why doesn't the stomach digest itself?. Physiol Rev.

[29] Allen A, Flemström G (2005). Gastroduodenal mucus bicarbonate barrier: protection against acid and pepsin. Am J Physiol Cell Physiol.

[30] Amagase K, Yokota M, Tsukimi Y, Okabe S (2003). Characterization of" unhealed gastric ulcers" produced with chronic exposure of acetic acid ulcers to indomethacin in rats. J Physiol Pharmacol.

[31] Potrich FB, Allemand A, da Silva LM, dos Santos AC, Baggio CH, Freitas CS (2010). Antiulcerogenic activity of hydroalcoholic extract of Achillea millefolium L: involvement of the antioxidant system. J Ethnopharmacol.

[32] Tan PV, Mezui C, Enow-Orock GE, Agbor G (2013). Antioxidant capacity, cytoprotection, and healing actions of the leaf aqueous extract of Ocimum suave in rats subjected to chronic and cold-restraint stress ulcers. Ulcers.

[33] Tarnawski AS (2005). Cellular and molecular mechanisms of gastrointestinal ulcer healing. Digest Dis Sci.

[34] Repetto MG, Llesuy SF (2002). Antioxidant properties of natural compounds used in popular medicine for gastric ulcers. Braz J Med Biol Res.

[35] Ali T, Harty RF (2009). Stress-induced ulcer bleeding in critically ill patients. Gastroenterology Clinics.

[36] Shaaban Azab K, Bashandy M, Salem M, Ahmed O, Tawfik Z, Helal H (2011). Royal jelly modulates oxidative stress and tissue injury in gamma irradiated male Wister Albino rats. N Am J Med Sci.

[37] Mc Tavish D, Buckly MM, Heel RC (1991). Omeprazole. An update review of its pharmology and therapeutic use in acid-related disordered. Drugs.

[38] Zamani Z, Reisi P, Alaei H, Pilehvarian A (2012). Effect of royal jelly on improving passive avoidance learning and spatial learning and memory in rats. J Shahid Sadoughi Univ Med Sci.

[39] Chai J, Baatar D, Tarnawski A (2004). Serum response factor promotes re-epithelialization and muscular structure restoration during gastric ulcer healing. Gastroenterology.

[40] El-Hady FK, El Awdan SA, Ibrahim AM (2013). Anti-ulcerative potential of Egyptian propolis against oxidative gastric injury induced by indomethacin in rats. Asian J Pharm Clin Res.

[41] Czarnecki R, Librowski T (2010). Effect of propolis on the healing of ethanol- and acetic acid-induced chronic gastric ulcer in rats. Acta Biol Cracov Zool.

[42] Ijioma SN, Nwaogazi EN, Nwankwo AA, Oshilonya H, Ekeleme CM, Oshilonya LU (2018). Histological exhibition of the gastroprotective effect of Moringa oleifera leaf extract. Comp Clin Pathol.

[43] Siavash M, Shokri S, Haghighi S, Shahtalebi MA, Farajzadehgan Z (2015). The efficacy of topical royal jelly on healing of diabetic foot ulcers: a double-blind placebo-controlled clinical trial. Int Wound J.

